# Medical Students' Exposure to and Attitudes about the Pharmaceutical Industry: A Systematic Review

**DOI:** 10.1371/journal.pmed.1001037

**Published:** 2011-05-24

**Authors:** Kirsten E. Austad, Jerry Avorn, Aaron S. Kesselheim

**Affiliations:** 1 Division of Pharmacoepidemiology and Pharmacoeconomics, Department of Medicine, Brigham and Women's Hospital and Harvard Medical School, Boston, Massachusetts, United States of America; 2 Edmond J. Safra Center for Ethics at Harvard University, Cambridge, Massachusetts, United States of America; York University, Canada

## Abstract

**Background:**

The relationship between health professionals and the pharmaceutical industry has become a source of controversy. Physicians' attitudes towards the industry can form early in their careers, but little is known about this key stage of development.

**Methods and Findings:**

We performed a systematic review reported according to PRISMA guidelines to determine the frequency and nature of medical students' exposure to the drug industry, as well as students' attitudes concerning pharmaceutical policy issues. We searched MEDLINE, EMBASE, Web of Science, and ERIC from the earliest available dates through May 2010, as well as bibliographies of selected studies. We sought original studies that reported quantitative or qualitative data about medical students' exposure to pharmaceutical marketing, their attitudes about marketing practices, relationships with industry, and related pharmaceutical policy issues. Studies were separated, where possible, into those that addressed preclinical versus clinical training, and were quality rated using a standard methodology. Thirty-two studies met inclusion criteria. We found that 40%–100% of medical students reported interacting with the pharmaceutical industry. A substantial proportion of students (13%–69%) were reported as believing that gifts from industry influence prescribing. Eight studies reported a correlation between frequency of contact and favorable attitudes toward industry interactions. Students were more approving of gifts to physicians or medical students than to government officials. Certain attitudes appeared to change during medical school, though a time trend was not performed; for example, clinical students (53%–71%) were more likely than preclinical students (29%–62%) to report that promotional information helps educate about new drugs.

**Conclusions:**

Undergraduate medical education provides substantial contact with pharmaceutical marketing, and the extent of such contact is associated with positive attitudes about marketing and skepticism about negative implications of these interactions. These results support future research into the association between exposure and attitudes, as well as any modifiable factors that contribute to attitudinal changes during medical education.

*Please see later in the article for the Editors' Summary*

## Abstract 2

A systematic review of published studies reveals that undergraduate medical students may experience substantial exposure to pharmaceutical marketing, and that this contact may be associated with positive attitudes about marketing.

## Editors' Summary

**Background:**

The complex relationship between health professionals and the pharmaceutical industry has long been a subject of discussion among physicians and policymakers. There is a growing body of evidence that suggests that physicians' interactions with pharmaceutical sales representatives may influence clinical decision making in a way that is not always in the best interests of individual patients, for example, encouraging the use of expensive treatments that have no therapeutic advantage over less costly alternatives. The pharmaceutical industry often uses physician education as a marketing tool, as in the case of Continuing Medical Education courses that are designed to drive prescribing practices.

One reason that physicians may be particularly susceptible to pharmaceutical industry marketing messages is that doctors' attitudes towards the pharmaceutical industry may form early in their careers. The socialization effect of professional schooling is strong, and plays a lasting role in shaping views and behaviors.

**Why Was This Study Done?:**

Recently, particularly in the US, some medical schools have limited students' and faculties' contact with industry, but some have argued that these restrictions are detrimental to students' education. Given the controversy over the pharmaceutical industry's role in undergraduate medical training, consolidating current knowledge in this area may be useful for setting priorities for changes to educational practices. In this study, the researchers systematically examined studies of pharmaceutical industry interactions with medical students and whether such interactions influenced students' views on related topics.

**What Did the Researchers Do and Find?:**

The researchers did a comprehensive literature search using appropriate search terms for all relevant quantitative and qualitative studies published before June 2010. Using strict inclusion criteria, the researchers then selected 48 articles (from 1,603 abstracts) for full review and identified 32 eligible for analysis—giving a total of approximately 9,850 medical students studying at 76 medical schools or hospitals.

Most students had some form of interaction with the pharmaceutical industry but contact increased in the clinical years, with up to 90% of all clinical students receiving some form of educational material. The highest level of exposure occurred in the US. In most studies, the majority of students in their clinical training years found it ethically permissible for medical students to accept gifts from drug manufacturers, while a smaller percentage of preclinical students reported such attitudes. Students justified their entitlement to gifts by citing financial hardship or by asserting that most other students accepted gifts. In addition, although most students believed that education from industry sources is biased, students variably reported that information obtained from industry sources was useful and a valuable part of their education.

Almost two-thirds of students reported that they were immune to bias induced by promotion, gifts, or interactions with sales representatives but also reported that fellow medical students or doctors are influenced by such encounters. Eight studies reported a relationship between exposure to the pharmaceutical industry and positive attitudes about industry interactions and marketing strategies (although not all included supportive statistical data). Finally, student opinions were split on whether physician–industry interactions should be regulated by medical schools or the government.

**What Do These Findings Mean?:**

This analysis shows that students are frequently exposed to pharmaceutical marketing, even in the preclinical years, and that the extent of students' contact with industry is generally associated with positive attitudes about marketing and skepticism towards any negative implications of interactions with industry. Therefore, strategies to educate students about interactions with the pharmaceutical industry should directly address widely held misconceptions about the effects of marketing and other biases that can emerge from industry interactions. But education alone may be insufficient. Institutional policies, such as rules regulating industry interactions, can play an important role in shaping students' attitudes, and interventions that decrease students' contact with industry and eliminate gifts may have a positive effect on building the skills that evidence-based medical practice requires. These changes can help cultivate strong professional values and instill in students a respect for scientific principles and critical evidence review that will later inform clinical decision-making and prescribing practices.

**Additional Information:**

Please access these Web sites via the online version of this summary at http://dx.doi.org/10.1371/journal.pmed.1001037.

- Further information about the influence of the pharmaceutical industry on doctors and medical students can be found at the American Medical Students Association PharmFree campaign and PharmFree Scorecard, Medsin-UKs PharmAware campaign, the nonprofit organization Healthy Skepticism, and the Web site of No Free Lunch.

## Introduction

The relationship between physicians and the pharmaceutical industry has become a major topic of concern for health services researchers [1] and policymakers [2], as well as in the lay media. While opinions about such relationships vary [3]–[6], it is clear that physicians have a high level of exposure to industry marketing in a variety of forms, which impacts clinical decision making [4].

Industry involvement in medical education occurs on multiple levels, including one-on-one meetings between trainees and pharmaceutical sales representatives (PSRs) and sponsored publications and educational events (such as Continuing Medical Education courses). Because pharmaceutical companies recognize the potential for education to be used as a marketing tool [7],[8], there is concern that such exposure may communicate a biased message encouraging overuse of particular products [9],[10]. Interactions with PSRs can increase prescriptions of the drug being promoted and shift prescribing in ways that may not be consistent with evidence-based guidelines [11]–[13]. One common outcome is the use of expensive treatments without therapeutic advantage over less costly alternatives [4],[14],[15]. Industry-sponsored education may also influence physicians' ability to weigh the risk-benefit profiles of new, heavily promoted drugs. For example, in the case of rofecoxib (Vioxx), pharmaceutical manufacturer–sponsored educational materials downplayed the drug's cardiac risks (a nearly 2-fold increased risk of heart attack and stroke) [16].

Why does pharmaceutical industry marketing have such a substantial effect on physician behavior [17]? One explanation may be that physicians' attitudes towards the industry and their propensity to be influenced by its marketing form very early in their careers. The socialization effect of professional schooling is strong [18]–[20], and plays a lasting role in shaping students' views and behaviors [21]. For example, a study examining the behavior of physicians trained in residency programs that limit contact with PSRs found that such policies shape subsequent decision making [22]. Therefore, encouraging more rational prescribing among practicing physicians may require a better understanding of how medical students interact with the pharmaceutical industry.

Moves to limit industry influence on undergraduate medical education have been contentious. In recent years, medical schools have taken proactive steps to limit students' and faculties' contact with industry [23]. These steps have included instituting guidelines for speaking and consulting relationships and mandating faculty disclosure of potential conflicts of interest on a public website [24],[25]. However, some have argued that these restrictions are detrimental to students' education and the future of biomedical research [26],[27].

Given the controversy over the pharmaceutical industry's role in undergraduate medical training, synthesizing the current state of knowledge is useful for setting priorities for changes to educational practices and the establishment of a research agenda. We systematically examined the peer-reviewed literature through May 2010 to collect empirical data quantifying medical students' exposure to and perspectives on pharmaceutical marketing practices, including their behaviors related to prescribing and attitudes about important drug policy topics. Specifically, we examined the extent of pharmaceutical industry interactions with medical students, whether such interactions influenced students' views on related topics, and whether any differences exist between students in their preclinical versus clinical years or in different learning environments in relation to these issues.

## Methods

### Data Sources and Searches

We searched MEDLINE (PubMed), EMBASE, Web of Science, and ERIC (EBSCOHost) for peer-reviewed articles from the earliest available dates through May 2010 with the help of a medical librarian. For search terms, two main subject heading domains were combined with the AND operator: one to designate the population (e.g., “medical students”) and the other to designate the topics relevant to the research question (e.g., “pharmaceutical industry” and “conflict of interest”). A full list of search terms is available in Table S1. Both Medical Subject Heading (MeSH) terms (or equivalent) and free text were utilized. No language requirement was placed on the search. Nine additional abstracts not captured by the search strategy were identified through review of the bibliographies of included articles.

### Study Selection

We developed a screening strategy using three criteria. First, studies were required to present data specific to medical students. If a study did not indicate whether the year of the students reflected clinical or preclinical training, this information was obtained from descriptions of the medical curricula on the institutional website(s) where the survey was conducted.

Second, studies had to include an observational or experimental design and employ quantitative or qualitative methods. We excluded editorials and other nonempirical opinion pieces. If the study reported pretests and post-tests related to an educational intervention, only preintervention data were analyzed (this occurred in six studies).

Finally, studies were required to report data on either (a) students' exposures to pharmaceutical industry marketing (e.g., counts of meetings with PSRs, gifts, and attendance at industry-sponsored educational events), or (b) students' knowledge, attitudes, and behaviors relating to industry, prescribing practices, or pharmaceutical policy issues, including the educational value of marketing materials, the costs of drug development or treatment regimens, and generic drug use. We excluded studies reporting students' perspectives on complementary and alternative medicines, use of specific therapeutic classes (such as antipsychotics), and medical errors and safety as long as those studies did not also examine industry marketing practices in relation to those topics.

Our screening criteria were applied separately in a pilot phase by two authors (KEA and ASK) on a selection of 10% of the pulled abstracts to ensure clarity of the criteria and reproducibility of the results. Then, one of us (KEA) reviewed the entire list of abstracts and identified articles for full review.

### Data Extraction and Quality Assessment

We noted the study type and characteristics of the populations studied, including year in medical school (preclinical versus clinical), country, sample size, and response rate. Next, we extracted primary data using a piloted extraction tool, including: exposure to industry (type of interaction and frequency); student attitudes about pharmaceutical marketing practices; views and practices related to evidence-based prescribing; and perspectives on use of generic drugs, drug development, and cost of treatment. We identified any correlations between measures (such as exposure and attitudes) and the methodology used to test the correlation. Non-English language articles were translated by a native speaker.

We assessed quality of survey studies using the Glaser and Bero protocol [28], a five-point scale for rating surveys based on study population, generalizability, survey content and construction, and data analysis. Other investigators have also used this strategy in systematic reviews of articles presenting survey data [29]. Two authors (KEA and ASK) independently rated each study and disagreements (which occurred in seven out of the 29 rated) were resolved by consensus.

### Data Synthesis and Analysis

Given the heterogeneity of studies, qualitative rather than quantitative synthesis of data was performed. We sorted studies on the basis of population training level: “preclinical” (defined as predominantly classroom education), “clinical” (defined as primarily clinical education, including clerkship), or “both.” Data regarding student attitudes were grouped according to type of marketing practice or industry relationship queried. We also performed a sensitivity analysis to explore the effect of excluding older studies (those performed before 2000) and those of lower methodological strength (score 0–2) from our results. The funders of the study played no role in the design of the study, data interpretation, or manuscript preparation. The PRISMA flowchart is available in Text S1.

## Results

**Figure 1:**
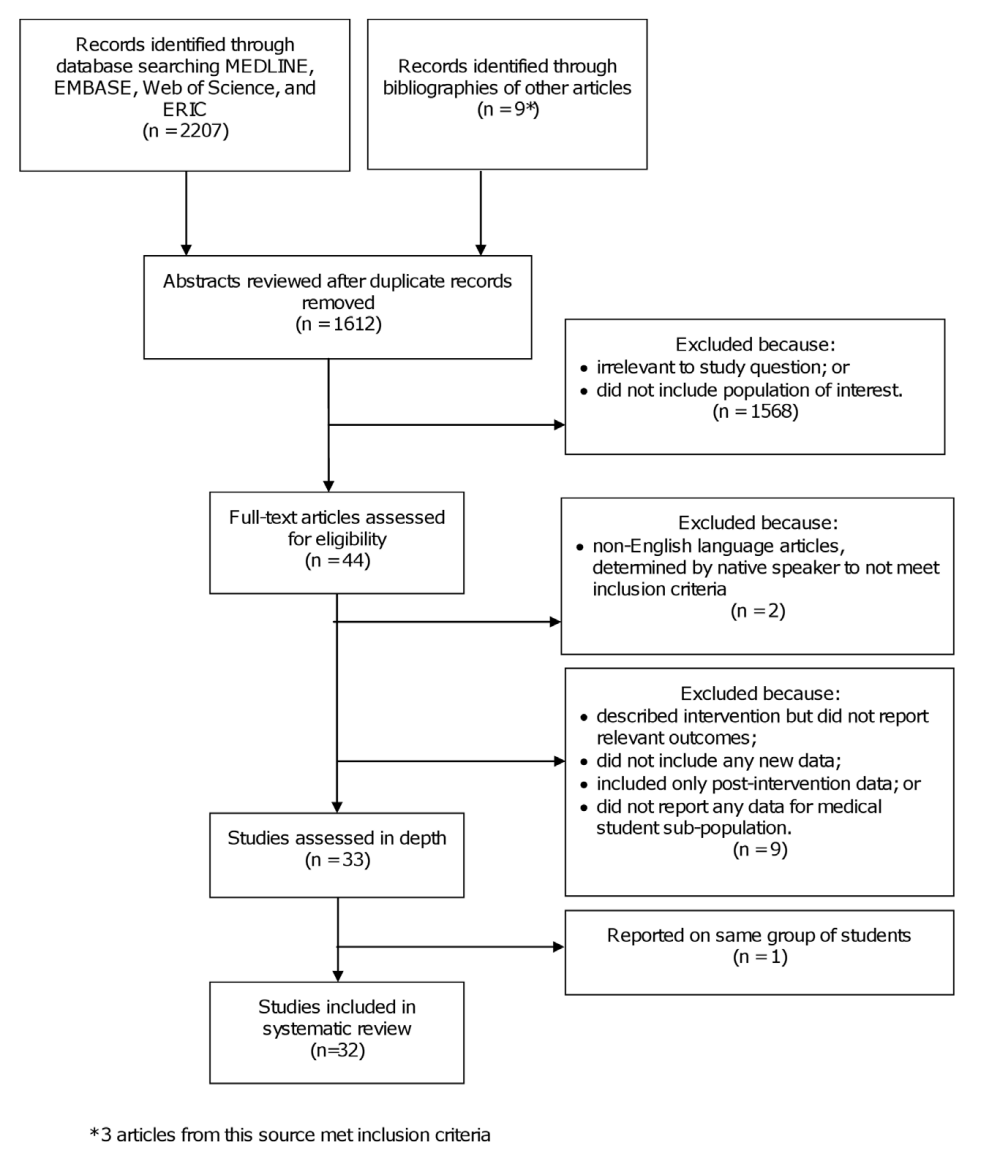
PRISMA schematic of systematic review search process.

Our search strategy produced 1,603 abstracts. We identified 48 articles for full review and confirmed 33 [30]–[62] as eligible for analysis (Figure 1) [63]. Two papers [46],[47] reported overlapping data from the same sample of students, so we combined them for an effective total of 32 studies. The vast majority of studies (29/32, 91%) [30]–[32],[34]–[36],[38]–[39],[41]–[62] used a cross-sectional survey as the primary methodology, occasionally supplemented with other techniques, such as informant interviews [54] and analyses of student journals [30]. The remaining study designs included a practical exam [33], a case study [40], and a randomized experiment [37]. In total, studies assessed approximately 9,850 medical students at 76 medical schools or hospitals (one study [49] did not specify participants' school affiliation). All studies reviewed are listed in Table 1.

**Table 1:** Empirical studies of medical students' attitudes about and exposures to pharmaceutical industry included in the systematic review.

First Author, Year (Country)	Primary Methodology	Response Rate	Quality Rating (out of 5)^a^	Main Findings
**Studies including only preclinical students**				
Sarikaya, 2009 (Turkey) [30]	Cross-sectional survey, multi-institutional	308/398, 77%	3	91% students experienced industry marketing. Favorable attitudes toward industry were more common for those who had interactions with PSRs (versus no interaction, OR = 2.974, *p* = 0.012)
Fein, 2007 (US) [31]	Cross-sectional survey	226/288, 79%	4	77% students had received gifts by their third semester, 24% agreed that accepting gifts would influence their future prescribing.
Ball, 2007 (Kuwait) [32]	Cross-sectional survey	103/299, 34%	3	70% reported that textbook is appropriate gift, 24% believed the same for meal. 74% believed that drug company presentations were biased.
Al Khaja, 2005 (Bahrain) [33]	Objective structured practical exam	539	N/A	81% of drugs correctly prescribed by students were written with generic instead of brand names
Vinson, 1993 (US) [34]	Cross-sectional survey (preintervention)	156/215, 73%	3	No observed difference in acceptance of marketing between 1st and 2nd years
**Studies including only clinical students**				
Lea, 2010 (Norway, Hungary, Poland) [35]	Cross-sectional survey, multi-institutional	819/1,245, 66%	4	74% students had contact with pharmaceutical industry. Exposure correlated with self-perceived ability to handle industry interactions.
Tichelaar, 2009 (Netherlands) [36]	Cross-sectional survey, multi-institutional	32/32, 100%	3	Students identified “effectiveness of the drugs” and “examples from medical teachers” as the most important factors in determining treatment choice.
Grande, 2009 (US) [37]	Randomized experimental design with follow-up survey assessment, multi-institutional	352	N/A	Students from school with policy limiting industry interactions had significantly less favorable attitudes about industry, including increased skepticism (mean scaled score: 0.42 versus 0.55, *p*<0.001)
Markham, 2009 (US) [38]	Cross-sectional survey (preintervention)	243	2	Around 95% reported that they accepted gifts from industry. Students estimated that the average drug costs US$20–US$50 million to develop.
Volodina, 2009 (Russia, Germany) [39]	Cross-sectional survey, multi-institutional	226/240, 94%	3	Nearly all students agreed that corporate social responsibility should be important for pharmaceutical industry
Tardif, 2009 (Canada) [40]	Case-study	17	N/A	23% students exposed to drug samples in previous year. 67% believed that samples increased use of non–first-line treatments.
Straand, 2008 (Norway) [41]	Cross-sectional survey	144/241, 60% (survey only)	4	Students most commonly received food (90%) and educational material (87%) from PSR interactions.
Hassali, 2007 (Australia) [42]	Cross-sectional survey, multi-institutional	400/1,497, 27%	4	Poor performance on test of criteria for generic drug bioequivalence. Respondents reported that generics had lower safety standards, produced more side-effects, and were less effective than brand-name drugs.
Sierles, 2005 (US) [43]	Cross-sectional survey, multi-institutional	826/1,143, 72%	5	On average interacted with industry once per week. Exposure correlated positively with acceptance and negatively with skepticism.
Wofford, 2005 (US) [44]	Cross-sectional survey (preintervention)	75	3	87% believed PSR information was biased; 44% agreed that PSRs impacted physician prescribing.
Stanley, 2005 (UK) [45]	Cross-sectional survey (preintervention)	29	1	Mean score on drug development test was 33%. Majority agreed that “Results of clinical studies rather than marketing influence doctor prescribing.”
Monaghan, 2003 (US) [46],[47]	Cross-sectional survey	59/108, 55%	3	Students interacted with PSRs on average 10.6 times per month. 40% students correctly estimated industry marketing expenditures.
Wilkes, 2001 (US) [48]	Cross-sectional survey (preintervention)	120	3	Every student received at least one gift from industry. 35% felt they were skilled at critically assessing promotional material.
Sandberg, 1997 (US) [49]	Cross-sectional survey, multi-institutional	205/205, 100%	0	90% students received ≥1 book from pharmaceutical company, 25% correctly recalled the specific company responsible.
Hodges, 1995 (Canada) [50]	Cross-sectional survey	17/21, 81%	3	41% agreed that PSRs had important teaching role. >50% students believed that PSRs had no impact on prescribing
Weber, 1986 (Canada) [51]	Cross-sectional survey	28/28, 100%	2	In estimating the cost of treatment regimens, medical students were most likely to correctly estimate (40%) or underestimate (40%) the actual cost
Palmisano, 1980 (US) [52]	Cross-sectional survey (preintervention)	100	1	85% believed it was improper for a public official to accept a gift; 46% reported it was improper for a medical students to do so (chi-squared, 2 df = 16.94, *p*<0.0001)
**Studies including both preclinical and clinical students**				
Vuorenkoski, 2008 (Finland) [53]	Cross-sectional survey, multi-institutional	1,523/∼2,700, 57%	5	17% clinical and 1% preclinical attended ≥2 PSR presentations per month. Industry-sponsored education was one source for learning.
Fabbri, 2008 (Italy) [54]	Cross-sectional survey	190/190, 100%	3	71% said that interaction with or gifts from PSR influenced a doctor, but only 24% said it affected their own behavior.
Fitz, 2007 (US) [55]	Cross-sectional survey, multi-institutional	667/DNP, 20%–80%^b^	4	28% of preclinical and 65% clinical students thought it was appropriate to accept gift (*p*<0.001). Level of knowledge about drug development was same for both groups.
Hyman, 2007 (US) [56]	Cross-sectional survey	418/723, 58%	3	18% believed that curriculum should include industry-sponsored events; 61% felt insufficiently educated on interactions with industry.
Vainiomaki, 2004 (Finland) [57]	Cross-sectional survey, multi-institutional	952/2,800, 34%	4	20% preclinical and 68% clinical students attended ≥2 PSR presentations per month. Pharmaceutical industry was identified as one source for learning.
Bellin, 2004 (US) [58]	Cross-sectional survey	221/281, 79%	3	Clinical students had significantly higher exposure to industry than preclinical for most types of interactions. Contact was most frequent in internal medicine setting.
Barfett, 2004 (Canada)^c^ [59]	Cross-sectional survey	202/372, 54%	3	Students found inexpensive gifts more acceptable. No difference was noted in attitudes by level of training.
Barry, 2000 (US)^c^ [60]	Cross-sectional survey	208/528, 39%	3	For the scenario of a pharmaceutical company paying a physician for each patient enrolled in a clinical research project, approximately 22% students chose the most appropriate professional behavior
Mantyranta, 1995 (Finland)^c^ [61]	Cross-sectional survey	126/161, 78%	1	70% students supported marketing of drugs; 48% supported existence of industry-sponsored social events
Barnes, 1971 (US) [62]	Cross-sectional survey	254^b^	2	70% supported no longer soliciting industry support for social activities. Acceptance of promotion increased with more training (no *p*-value).

Response rate or number of participants was calculated if number not provided in article. A label of “multi-institutional” indicates studies that included students from more than one medical school or hospital.

a Rating based on a 5-point scale developed by Glaser and Bero [28].

b Number of potential participants and overall response rate were not reported.

c These studies included preclinical and clinical medical students in their study, but did not present any data separately to allow for comparison between these two groups.

DNP, did not provide; N/A, not applicable.

The studies included in this review were published between 1971 and 2010; however, only seven (7/32, 22%) were published before 2000, and the majority of these (5/7, 71%) received a score of 0, 1, or 2 for methodological quality. Over half assessed medical students from the US (15/32, 47%) or Canada (4/32, 13%), but Australia, Russia, and countries in Europe and the Middle East were also represented. Nearly all employed a self-report cross-sectional survey design; many employed additional qualitative methodologies including free-text response, focus groups, and analysis of student journal entries. Seventeen (53%) evaluated only clinical students, five (16%) preclinical students, and ten (31%) compared clinical and preclinical students. Sample sizes ranged from 17 to 1,523. The median methodological quality score was 3 out of 5 (interquartile range = 2–4).

### Exposure to Pharmaceutical Marketing

Medical students reported frequently interacting with the pharmaceutical industry (Table 2). Common types of interactions include involved gifts [31],[32],[40],[43],[48]–[50],[55],[58], industry-sponsored educational sessions [43],[53],[57], and direct communications with sales representatives [30],[41],[44],[46],[47],[50],[53],[55],[57]. We found that 89%–98% of students in the clinical years reported having accepted a lunch or snack provided by the pharmaceutical industry [43],[58]; one study of clinical students reporting on interactions with PSRs reported that 90% of exchanges involved food [41]. One multi-institution study from 2005 calculated that third-year American medical students interacted with industry on average once per week [43]. Up to 90% [41],[43],[49] of surveyed students in their clinical years had received educational materials such as textbooks or journal reprints from industry. Substantial variability was noted between studies performed in different countries, with the highest level of exposure occurring in the US, including two studies [48],[58] that found 100% of students had at least one interaction.

**Table 2:** Exposures of medical students to the pharmaceutical industry.

Type of Exposure	Percentage of Preclinical Students Reporting Interaction^a^	Percentage of Clinical Students Reporting Interaction^a^
Any interaction	61% [32]; 91% [30]; 97% [58]	74% [35]; 100% [48]; 100% [58]; 1/week (since start of clerkship) [43]
Interaction with PSRs	40% [55]; 64% [30]; 1% (at least 2/month) [53]; 20% (at least 2/month) [57]	95% [44]; >80% [55]; 17% (at least 2/month) [53]; 68% (at least 2/month) [57]; 10.6/month [46],[47]
Industry-sponsored educational events	0% (at least 2/month) [53]; 7% (at least 2/month) [57]	3% (at least 2/month) [53]; 22% (at least 2/month) [57]; 26% (since start of clerkship) [43]
Gifts	<40% [55]	80% [55]
• Fine dining or dinner		35% [48]; 51% [43]
• Other food	4% [32]; 11% (1st years) [31]; 53% (2nd years) [31]; 90% [58]	90% [41] b; 98% [58]; 89% (snack, since start of clerkship) [43]; 97% (lunch, since start of clerkship) [43]
• Noneducational gift (pen, mug)	18% (1st years) [31]; 57% (2nd years) [31]; 34% [32]; 63% [58]	44% [41] b; 92% [58]; 94% (since start of clerkship) [43]; 95% [48]
• Textbook/educational material	11% [32]; 11% (textbook) [58]; 30% (pocket text) [58]	26% (textbook) [58]; 51% (since start of clerkship) [43]; 68% [48]; 79% (pocket text) [58]; 87% [41] b
• Journal reprint/glossy handout	4% (1st years) [31]; 46% (2nd years) [31]; 42% [32]; 14% [59]	90% (since start of clerkship) [43]; 14% [59]
• Drug sample	1% (1st years) [31]; 11% (2nd years) [31]; 25% [32]	23% (during last year) [40]; 41% [38] b; 42% (since start of clerkship) [43]; 43% [48]
• Social event	5% [32]	34% (since start of clerkship) [43]

Each entry reports data on exposure of preclinical and clinical medical students from the studies included in our sample. Data from studies performed before 2000 or those that received a score of 0–2 on the Glaser-Bero scale are not included [34],[38],[45],[49]–[52],[61],[62].

a Data indicate students reporting at least one interaction during medical school (unless otherwise specified).

b Study sample derived from students who had at least one interaction with PSR and reported on one such interaction.

Overall, contact with the pharmaceutical industry increased over the course of medical school. This trend was observed both in studies reporting cumulative incidence (total number of exposures since starting medical school) in preclinical and clinical populations [30]–[32],[35],[38],[44],[48],[49],[55],[58], as well as studies considering exposure during a single academic year or per month [40],[43],[46],[47],[50],[53],[57]. This increase was consistent across most of the types of interactions listed in Table 2.

### Attitudes about Marketing Practices

Students' attitudes about pharmaceutical marketing practices were variable and occasionally contradictory (Table 3). Many students approved of meals [31],[32],[35],[43],[46],[47],[59], small promotional items [32],[43],[46],[48],[59],[62], and gifts with an educational purpose [31],[32],[35],[43],[46]–[48],[59], but were less accepting of social events [31],[32],[43],[61],[62] and travel [31],[32],[35],[43],[46]–[48]. However, 75% of students in an Italian study said they would renounce gifts from industry [54]. Students justified their entitlement to gifts by citing financial hardship (48%–80%) [31],[37],[43] or by asserting that most others accepted gifts [54].

**Table 3:** Attitudes of preclinical and clinical medical students toward physician–industry interactions.

Statements Describing Physician–Industry Interactions		Data Relating Agreement of Preclinical Students with Statements	Data Relating Agreement of Clinical Students with Statements
Topic	Statements		
General information/promotion	Is useful to learn about drugs	29% [31]; 62% [32]	53% [37]; 65% [37]; 71% [43]
	Has educational value		66% [35]; 49% [48]
	Influences own prescribing	11% [53]; 19% [57]	12% [53]; 25% [57]
	Does not influence own prescribing	LS 3.0 out of 5 [30]	74% [35]
	Is unethical	29% [32]	—
Pharmaceutical sales representatives	Feel PSRs should be excluded from learning environment	29% [31]; 26% [32]	67% [37]; 18% [37]; 17% [43]; LS 1.6 out of 5 [46],[47]
	Desire more interaction with PSRs	35% [57]; 40% [53]	24% [57]; 35% [53]
	Feel PSRs have important teaching role	39% [32]	LS 2.8 out of 5 [46],[47]
	Have educational value, or impart useful and accurate information	LS 2.6 out of 5 [30]	22% [44]; 4.2 out of 10 [41]; LS 3.1 out of 5 [46],[47]
	Are biased	—	87% [44]
	Provide trustworthy information	21% [32]	—
	Influence physician prescribing	LS 3.4 out of 5 [30]	44% [44]
	Do not influence own prescribing	—	LS 2.8 out of 5 [46],[47]
	Are bad for patients	LS 3.0 out of 5 [30]	—
Industry-supported grand rounds/educational presentations	Are biased	74% [32]	92% [37]; 68% [37]; 67% [43]
	Are useful/helpful/educational	36% [32]	52% [37]; 86% [37]; 89% [43]
	Desire more	46% [57]; 51% [53]	57% [57]; 56% [53]
	Should not be allowed	—	45% [35]
Gifts	Given to students		
	Are appropriate to accept	28% [55]; LS 1.7 out of 5 [56]	65% [55]; LS 1.5 out of 5 [56]
	Have minimal influence	34% [31]; 45% [32]	30% [37]; 61% [37]; 71% [43]
	Support because of minimal income	48% [31]	52% [37]; 74% [37]; 80% [43]
	Should not be restricted	—	24%–28% [44]
	Given to physicians		
	Are appropriate	30% [55]	>50% [55]
	Are inappropriate/unethical	—	3%–26% [48]
	Are inappropriate for government official	85% [55]	84% [55]
	Influence prescribing		
	Own	24% [31]; 33% [32]	63% [37]; 29% [37]; 31% [43]
	Other student	39% [31]	69% [37]; 38% [37]; 42% [43]
	Physician	—	13%–18% [48]
	Do not influence physician prescribing	70% [55]	72% [55]
	Do not influence own prescribing	—	LS 3.5 out of 5 [46],[47]
Drug Samples	Support because go to uninsured/needy	LS 3.4 out of 5 [30]	88% [40]
Education	Educated adequately on interactions with industry	11% [53]; LS 1.2 out of 5 [56]	LS 2.6 out of 5[46],[47]; 39% [53]; LS 1.7 out of 5 [56]
	Believe not sufficiently educated on interactions with industry	89% [53]	61% [53]; 83% [43]
	Feel competent to navigate interactions	—	41% [35]
	Desire more education	77%–79% [31]; 66% [53]	86% [35]; 78% [43]; 62% [53]
Disclosure	Support prelecture disclosure of potential conflicts	69% [31]	77% [35]
Faculty relationships with industry	Agree not ethical to receive research funds	—	12% [48]
	Agree not ethical to receive honoraria for lecturing	—	11%–12% [48]
	Feel acceptable to receive honoraria for lecturing	—	18% [35]
Institutional relationships and policies	Support industry funds to:		
	Lower tuition	26% [31]	44% [37]; 55% [37]; 54% [43]
	Pay for printing (with logo)	33% [31]	
	Believe adequate separation between teaching institution and pharmaceutical industry	LS 2.2 out of 5 [56]	81% [35]; LS 1.9 [56]
	Support industry-sponsored events in curriculum	LS 1.4 out of 5 [56]	LS 1.2 out of 5 [56]
	Agree should be regulated (by school or government)	LS 2.7 out of 5 [56]; LS 3.9 out of 5 [30]	LS 2.6 out of 5 [56]

This is a summary of the attitudes of preclinical and clinical medical students from the studies that met inclusion criteria. Likert scale (LS) data were all adjusted to standard of 1 = strongly disagree; 5 = strongly agree. For 10-point scales, 10 represents “very good.” Grande et al. [34] presented data for two distinct groups of medical students. Data from studies performed before 2000, those that received a score of 0–2 on the Glaser-Bero scale [34],[38],[45],[49]–[52],[61],[62], and those that did not report responses separately for preclinical and clinical populations [54],[59] in the relevant domains are not included.

When asked about the appropriateness of accepting gifts from industry overall, students at different levels of training expressed divergent opinions. In most studies, the majority of students in their clinical training years found it ethically permissible for medical students to accept gifts from drug manufacturers [37],[43],[52],[55],[56], while a smaller percentage (28%–48%) of preclinical students reported such attitudes [31],[32],[55],[56]. This same trend was seen in student opinion regarding whether physicians should accept gifts [48],[55]. Many students displayed exceptionalism with regard to the medical profession, as approximately 85% reported that it would be inappropriate for a government official to accept similar gifts [52],[55]. Two surveys found no change in perceived appropriateness of gifts from industry as students progressed in their training [32],[34].

One of the most consistently held student attitudes was the belief that education from industry sources is biased [32],[37],[43],[44], especially among clinical students (67%–92%) [37],[43],[44]. Despite this, students variably reported (22%–89%) that information obtained from industry sources was useful and a valuable part of their education [30]–[32],[35],[37],[43],[44],[46]–[48],[50], with clinical students more frequently endorsing the utility.

In most studies, almost two-thirds of students reported that they were immune to bias induced by promotion [53],[57], gifts [31],[32],[37],[43],[46],[47], or interactions with sales representatives in general [46],[47],[54]. This perception of immunity to bias was prevalent in both the preclinical and clinical years. It appeared that students were more likely to report that fellow medical students (38%–69%) or doctors (13%–71%) are influenced by such encounters than they were personally (24%–63%) [31],[37],[43],[46],[47],[54].

### Effect of Marketing Practices on Attitudes

Eight studies reported a relationship between exposure to the pharmaceutical industry and positive attitudes about industry interactions and marketing strategies (though not all included supportive statistical data) [30],[32],[35],[43],[49],[50],[53],[57]. In a national survey, students' overall level of exposure to pharmaceutical marketing was inversely correlated with the attitude that these interactions were inappropriate (*r* = −0.155; *p*<0.001) and with the belief that these educational sources were biased and influenced prescribing (*r* = −0.171; *p*<0.001) [43]. Students who interacted with PSRs were more likely than those who did not meet with PSRs to report positive perceptions of industry marketing (odds ratio [OR] = 2.974, *p* = 0.012) and were less likely to perceive this marketing as negative (OR = 0.408, *p* = 0.004) [30]. Lea et al. found that degree of industry exposure was associated with students' attitudes that they had the ability to self-regulate interactions with industry (31% versus 41% versus 50% versus 60%, *p*<0.001) and with the belief that accepting meals from industry was appropriate (82% versus 67%, *p*<0.001) [35]. As with all correlational studies, these data cannot demonstrate causation. A study of medical students, physicians, and other health professionals found a relationship between the number of gifts received and the belief that sales representatives do not influence prescribing (*r*_s_ = 0.24; *p*<0.04) [50], although this comes from one of the older studies in our sample and data specific to the medical student subgroup were not provided. Only one study found no relationship between students' total number of previous contacts with PSRs and perception of the educational value of PSR interactions (ANOVA *p* = 0.08) [44].

Students in different learning environments had significant differences in their reported attitudes [35],[37],[43],[53] with perspectives generally consistent with the policies of their schools. One randomized controlled trial exposed students to small promotional items and found differences in implicit attitudes between fourth-year students at two different schools that differed in the strength of their institutional policies regarding industry access [37]. In one national sample, the subset of students participating in clinical clerkships at hospitals that restricted direct industry marketing had less exposure to industry, according to mean exposure index (a measure of number of interactions experienced during a month of clerkship; 2.5 versus 4.6; *p*<0.001). On a skepticism scale derived from six of the survey questions (range, 0–1; mean skepticism score = 0.43), these students also displayed a significantly higher level of skepticism about marketing messages (mean skepticism score 0.45 versus 0.43; *p* = 0.03) [43]. A separate study found significant differences in attitudes regarding pharmaceutical marketing between students at two medical schools (mean skepticism score 0.55 versus 0.42; *p*<0.001) and attributed this divergence to the presence of restrictive policies present at one of the schools (with more skeptical attitudes expressed by these students) [37]. After a national reform limiting pharmaceutical marketing in clinical settings, the percentage of Finnish medical students who believed that marketing would influence their future clinical decisions decreased significantly [53].

### Attitudes on Reform

In the studies we identified, students generally did not support excluding sales representatives [31],[32],[37],[43],[46],[47] or industry presentations [35] from the learning environment. Student opinions were split on whether physician–industry interactions should be regulated by medical schools or the government; surveys from Italy and Kuwait reported more support for rule-setting than a US study [32],[54],[56]. Eighty-six percent of American medical students reported that during their residencies they would like to interact with PSRs (86%) [48], and two Finnish surveys [53],[57] found that 24%–57% of students wanted more industry-sponsored education. Faculty disclosure of conflicts of interest before lecturing was endorsed by 69%–77% of students across all studies [31],[35].

Most medical students reported not feeling adequately educated on physician–industry interactions [43],[46],[47],[53],[54],[56] with 62%–86% requesting more instruction in this area [31],[35],[43],[53],[54]. While 39% of clinical students reported being adequately educated on the topic, only 11% of preclinical students reported that the amount of instruction they received was sufficient [53].

### Other Pharmaceutical Policy Issues

The pharmaceutical industry was identified as one source of information used by students to learn about therapeutics (16%–49%) [46],[47],[53],[57]. But in one study, students who had interacted with a PSR reported that side effects, interactions, and contraindications of the promoted therapy were either not discussed or inadequately covered in these encounters [41].

Medical students reported little knowledge of drug costs or spending on pharmaceutical marketing [38],[45],[46],[47],[55], except in one survey of Italian medical students, in which 62% were knowledgeable [54]. Two surveys found no change in knowledge about these areas over the course of undergraduate medical training [54],[55]. When asked to estimate the actual cost of treatment described in six clinical scenarios, students underestimated the actual cost in 40% cases, which was similar to the responses of residents or attending physicians [51]. However, this study had methodological flaws and was conducted in 1986; we did not locate more recent studies to confirm this observation.

One study found that knowledge regarding generic medications was poor overall [42]. Students reported negative attitudes about generic drugs, with nearly all agreeing that they were less effective (95%) and of inferior quality (94%), and caused more side effects (93%) than branded drugs. However, in another study evaluating behavior, students from Bahrain tended to prescribe drugs more frequently using their generic name [33].

### Sensitivity Analysis

The oldest [34],[49]–[52],[61],[62] and lowest-quality studies [38],[45],[49],[51],[52],[61],[62]—a total of 9 studies—amounted to a total of 8 data points in our analysis (4.2% of the total number of data points). These data were used for supportive purposes only and the results of these studies are not included in Tables 2 and 3.

## Discussion

This comprehensive systematic review of medical students' interactions with the pharmaceutical industry found that students are frequently exposed to pharmaceutical marketing, even in the preclinical years when learning is mostly done in the classroom setting. However, we also found that the extent of students' contact with industry is associated with positive attitudes about marketing and skepticism about any negative implications of these interactions. These findings are compatible with the results of a more limited review [64] that examined PubMed-listed English language studies of medical student surveys related to pharmaceutical industry marketing. The year of training and the presence of policies restricting drug industry interactions with trainees appear to influence students' attitudes about the role of marketing and other important pharmaceutical policy issues.

Students' opinions about the pharmaceutical industry differed between the preclinical and clinical years. Compared with preclinical students, those in their clinical years reported more educational value in industry-provided material [31],[32],[35],[41],[44],[46]–[48],[50] and were more accepting of gifts from industry [37],[43],[48],[52],[54],[55],[56]—both to themselves and to professional physicians [31],[32],[55],[56]. Long hours spent working and studying and increasing financial hardship [65] may have contributed to these feelings of entitlement. Preclinical students were less likely to feel sufficiently educated on the topic of physician–industry interactions with the pharmaceutical industry [53],[56], though confidence on this topic was also uncommon among clinical students [43],[46],[47],[53],[54],[56].

Some evidence showed that student opinions varied by medical school and the extent of industry interactions in those communities. Sierles et al. observed that students placed at hospitals with policies limiting interactions with PSRs expressed significantly more critical views of industry than the other students surveyed, though it is not clear whether self-selection played a role [43]. Similar differences were found by Grande et al., with clinical students at the school with a strong policy regarding student-industry interactions differing in their attitudes with students at a school without a strong policy and as compared to the findings of Sierles et al. [37]. Few studies rigorously evaluate whether observed changes in attitude over the course of medical or among different learning environments are causal or simply correlational; this represents a significant limitation of the current literature.

Why would attitudes change over the course of medical education, or why do they differ between two groups of clinical students at different schools? One possible explanation is that industry representatives are effective in directly molding medical students' attitudes about these issues. Another possibility is that the characteristics of medical students' learning environments shape attitudes about the pharmaceutical industry. The implicit lessons communicated through institutional policies and role models have been described as the “hidden curriculum” by scholars of learning theory [66],[67]. The importance of role modeling is explicitly recognized, as students reported “examples from medical teachers” as one important influence on their prescribing decisions [36]. This socialization process has been implicated in other attitudinal changes seen over the course of medical training, such as cynicism [68], burnout [69], and lack of interest in primary care [70].

A number of features of medical education may potentiate these educational cues. First, students are rapidly developing a professional identity and forming a foundation of professional values, making it likely that they will absorb the norms of their surroundings in creating these attitudes. Second, their behavior is constrained by their position at the bottom of the social hierarchy. For example, one study found that 93% of third-year students had been asked or required to attend an industry-sponsored lunch by a superior [43]. This dynamic may help explain why students are likely to accept gifts from pharmaceutical industry representatives even if they believe it is inappropriate. Passive adoption of the norms displayed by role models and actions in contrast to personal values contribute to the socialization of medical students and may in turn impact their professional practice.

Medical students' attitudes in some domains were similar to those reported by residents and practicing physicians. We found that students were more approving of small gifts from industry and those said to have an educational purpose, as compared to large gifts [30]–[32],[34],[35],[46],[47],[54],[59],[62]. In a prior review, Wazana observed a similar pattern in residents and physicians [4]. However, other attitudes appeared to evolve over the course of medical education and practice. For example, more medical students in our analysis reported believing that gifts influence prescribing (24%–63%) [31],[32],[37],[43],[46]–[48],[54] than did practicing physicians in the Wazana review (8%–13%, Likert scale [LS] 1.6–1.8) [4]. Shifts in attitude that occur during the course of training may be attributable to clinicians' greater confidence in their ability to objectively evaluate scientific evidence and distinguish credible information from overstatements in marketing messages. Practicing physicians, however, have been found to be far less adept at this skill than they report [4],[12]. Thus, medical school may be an optimal time to educate about problematic issues associated with learning about drugs through pharmaceutical marketing channels.

Our study has several limitations. Most of the included studies were cross-sectional surveys, which have typical limitations of sampling response rate (representativeness and size), and the difficulty of imputing longitudinal change from cross-sectional data. The heterogeneity of survey questions made it impossible to combine results into a formal meta-analysis because of the risk of false-positive conclusions [71]. Nonetheless, we took steps to address the limitations of a narrative synthesis, such as introduction a formal grading system of each study's methodological strength. Our sensitivity analysis confirmed that the results reported are driven by the newest and highest quality studies identified. Since variability in phrasing of survey questions was common, we took a conservative approach to categorizing responses and reporting response ranges. Publication bias could have also impacted our conclusions.

Since relationships between the pharmaceutical industry and organized medicine are context dependent, some variability could be an effect of country or year of study that was not captured by analysis of the learning environment. We noted some cross-cultural similarities and differences in exposures and attitudes, but none of the included studies were designed specifically to address this issue and more robust data are needed. Likewise, some surveys did not account for confounders within the learning environment that could be important in shaping students' exposures and attitudes or secular trends. For instance, while most studies did not consider gender differences, one found that women were less willing to accept gifts from industry [54]. Future longitudinal surveys following individual trainees could more clearly map the trajectory of beliefs toward the pharmaceutical industry and related issues over the course of professional development and determine which characteristics (institutional, environmental, and personal) most strongly impact this process.

Despite these limitations, this review of the literature provides important insights into the nexus between the pharmaceutical industry and undergraduate medical education and in our view helps elucidate an agenda for moving forward. Our findings demonstrate a significant hole in the existing research, most notably the need for studies that can determine whether changes in student attitudes toward the pharmaceutical industry are caused by contact with industry sources, the influence of role models, institutional policies, or other factors.

Our review also is relevant to those who teach medical students, including those outside of the US (given the diversity of settings of the studies analyzed). Strategies to educate students on physician–industry interactions should directly address misconceptions about the effects of marketing and other biases that can emerge from industry interactions. Support for reforms such as prelecture disclosure of relevant faculty relationships with industry are likely to be well received by students. However, education alone may be insufficient if policymakers are not also engaged. Modifiable institutional characteristics, including rules regulating industry interactions, can play an important role in shaping students' attitudes. Interventions that decrease students' contact with industry and eliminate gifts may have a positive effect on building the “healthy skepticism” that evidence-based medical practice requires. Given the potential for educational and institutional messages to be counteracted by the hidden curriculum, changes should be directed at faculty and residents who serve as role models for medical students. These changes can help move medical education a step closer to two important goals: the cultivation of strong professional values, as well as the promotion of a respect for scientific principles and critical review of evidence that will later inform clinical decision-making and prescribing practices.

## Supporting Information

Table S1 **Systematic review search strategy.** The following search strategy was employed for searching PubMed and was adapted for other database; MeSH, medical subject headings.
(DOC)

Text S1 **PRISMA checklist.**
(DOC)

## Abbreviations

OR: odds ratio
PSR: pharmaceutical sales representative
