# Supplementary material for: Medical Students' Exposure to and Attitudes about the Pharmaceutical Industry: A Systematic Review
Source: PLoS Med. 2011 May 24;8(5):e1001037. doi: 10.1371/journal.pmed.1001037 (PMC3101205; doi:10.1371/journal.pmed.1001037)
Supplement: Table S1 — Systematic review search strategy. The following search strategy was employed for searching PubMed and was adapted for other database; MeSH, medical subject headings. (DOC) [file pmed.1001037.s001.doc]

**Table S1. Systematic review search strategy**

| **Population** | | **Key terms** | |
| --- | --- | --- | --- |
| MeSH terms | Free text | MeSH terms | Free text |
| “education, medical, undergraduate,” “students, medical,” “clinical clerkship” | “undergraduate medical education,” “medical students,” “clerk,”  “clerkship” | “drug industry,” “marketing,” “drug discovery,” “drugs, generic,” “drug approval,” “conflict of interest,” “organizational policy,” “drug prescriptions,” “drug utilization,” “pharmacology, clinical/education,” “drug therapy” AND “attitude of health professional,” “evidence-based medicine” AND “drug therapy” | “pharmaceutical industry,” “drug industry,” “drug company,” “pharmaceutical representative,” “pharmaceutical marketing,” “pharmaceutical promotion,” “drug development,” “drug discovery,” “trade name,” “generic*,” “conflict of interest,” “medical school policy,” “industry interaction*,” “prescription,” “prescribing,” “pharmacology education,” “rational prescribing,” “evidence-based prescribing,” “drug approval,” medication AND attitude, “evidence-based medicine” AND medication |

The following search strategy was employed for searching PubMed and was adapted for other database; MeSH = medical subject headings.
